# P53 and Rb Aberrations in Small Cell Lung Cancer (SCLC): From Molecular Mechanisms to Therapeutic Modulation

**DOI:** 10.3390/ijms25052479

**Published:** 2024-02-20

**Authors:** Kostas A. Papavassiliou, Amalia A. Sofianidi, Vassiliki A. Gogou, Nektarios Anagnostopoulos, Athanasios G. Papavassiliou

**Affiliations:** 1 First University Department of Respiratory Medicine, ‘Sotiria’ Hospital, Medical School, National and Kapodistrian University of Athens, 11527 Athens, Greece; gogvanessa@gmail.com (V.A.G.); aris.anag@yahoo.gr (N.A.); 2 Department of Biological Chemistry, Medical School, National and Kapodistrian University of Athens, 11527 Athens, Greece; amsof.00@gmail.com

**Keywords:** p53, Rb, small cell lung cancer, genetic alterations and aberrations, mutations

## Abstract

The genes coding for the tumor suppressors p53 and retinoblastoma (Rb) are inactivated in the vast majority of small cell lung cancer (SCLC) tumors. Data support the notion that these two deleterious genetic events represent the initial steps in the development of SCLC, making them essential for a lung epithelial cell to progress toward the acquisition of a malignant phenotype. With the loss of *TP53* and *RB1*, their broad tumor suppressive functions are eliminated and a normal cell is able to proliferate indefinitely, escape entering into cellular senescence, and evade death, no matter the damage it has experienced. Within this setting, lung epithelial cells accumulate further oncogenic mutations and are well on their way to becoming SCLC cells. Understanding the molecular mechanisms of these genetic lesions and their effects within lung epithelial cells is of paramount importance, in order to tackle this aggressive and deadly lung cancer. The present review summarizes the current knowledge on p53 and Rb aberrations, their biological significance, and their prospective therapeutic potential, highlighting completed and ongoing clinical trials with agents that target downstream pathways.

## 1. Introduction

Cancer development and progression are multifaceted processes that entail several steps and rely on the persistent expression/silencing or activation/inactivation of transcription factors to facilitate the malignant growth and survival of cancer cells [1]. Transcription factors are DNA-binding proteins that attach to specific promoter or enhancer DNA sequences to orchestrate gene transcription by physically interacting with RNA polymerase and transcriptional co-regulators, including co-activators and co-repressors. Small cell lung cancer (SCLC) is a highly aggressive and lethal neuroendocrine lung cancer characterized by transcriptional deregulation, with mutations in the well-known transcriptional regulators p53 and retinoblastoma (Rb) being critical for its development [2].

The *TP53* and *RB1* tumor suppressor genes, responsible for encoding the transcriptional regulators p53 and Rb, respectively, are almost universally inactivated in SCLC cells and are considered the genomic hallmarks of SCLC [3]. This review summarizes recent advances in our understanding of p53 and Rb aberrations, their functional significance in the pathobiology of SCLC development and progression, and their potential clinical application, emphasizing completed and ongoing clinical trials with various therapeutic agents that target dysregulated downstream signaling pathways.

## 2. P53 and Rb Inactivation in SCLC

Loss of p53 and Rb protein function is strongly associated with cancer biology, occurring in a plethora of cancers [4,5,6,7]. p53 is a major tumor suppressive transcription factor that responds to several cellular stresses, including DNA damage, oxidative stress, hypoxia, lack of nutrients, and hyperproliferative stimuli. Physiologically, the protein levels of p53 are under the control of mouse double minute 2 homolog (MDM2), a nuclear-localized E3 ubiquitin ligase, which triggers the breakdown of p53 through the addition of ubiquitin tags, i.e., ubiquitylation, and subsequently, proteasomal degradation. p53 and MDM2 form a negative feedback loop, whereby p53 upregulates the protein expression of MDM2 by binding to the latter’s promoter, hence inducing p53 protein breakdown and keeping low levels of p53 within the cell [8]. When cellular stresses are present, p53 becomes phosphorylated and acetylated, leading to its dissociation from MDM2, its stabilization, and accumulation in the cell nucleus, where it is potentiated and initiates the transcriptional activation or repression of its target genes that are responsible for halting the proliferation of genomically damaged cells [9]. The transcriptional activity of p53 upregulates the expression of numerous genes that participate in DNA repair mechanisms, cell cycle arrest, cell apoptosis, autophagy, and cellular senescence, all of which aim to maintain the stability of the cellular genome and suppress cancer development [10,11,12]. Therefore, cells that harbor loss-of-function mutations in the gene *TP53* are unable to repair DNA damage and acquire potentially oncogenic genetic alterations, cannot enter senescence and undergo apoptosis in response to oncogenic signals, and proliferate excessively and uncontrollably.

Rb is another important transcriptional regulator with tumor suppressive functions that is involved in the regulation of the progression of the cell cycle [13,14]. It exerts its regulatory function via forming protein complexes with the E2F family of transcription factors and binding to the promoters of genes that trigger progression through the S phase of the cell cycle and cell proliferation. The physical interaction of Rb with E2F transcription factors attracts transcriptional co-repressors to these gene promoters or impedes the binding of transcriptional co-activators, either way resulting in downregulation of the protein expression of the cell cycle-related genes and blocking the transition from the G1 phase to the S phase of the cell cycle. When upstream mitogenic signals are present, cyclin-dependent kinases (CDKs), including CDK2, CDK4, and CDK6, associate with cyclins, including cyclin D and cyclin E, and once activated they go on to phosphorylate Rb [15,16]. The addition of multiple phosphate groups generates a hyperphosphorylated Rb protein that releases E2F transcription factors and allows the recruitment of transcriptional co-activators, thus promoting the transcription of S phase-related genes, which was previously repressed by Rb. As the cell progresses through the S phase of the cell cycle, the activity of CDKs is reduced and enzymes that remove phosphate groups, such as protein phosphatase 1, dephosphorylate Rb, returning the latter to a state where it can reform the Rb–E2F complexes that downregulate the protein expression of DNA synthesis and cell cycle progression genes [17]. Given the central role of Rb in the negative regulation of the cell cycle, it becomes evident why loss-of-function mutations in the gene *RB1* renders cells capable of transforming into cancer cells. In most cancers, mutations affect the *RB1* gene or genes coding for components of the Rb signaling pathway, for example, CDK4, CDK6, cyclin D, or the inhibitor of CDKs protein p16 [18].

**Figure 1:**
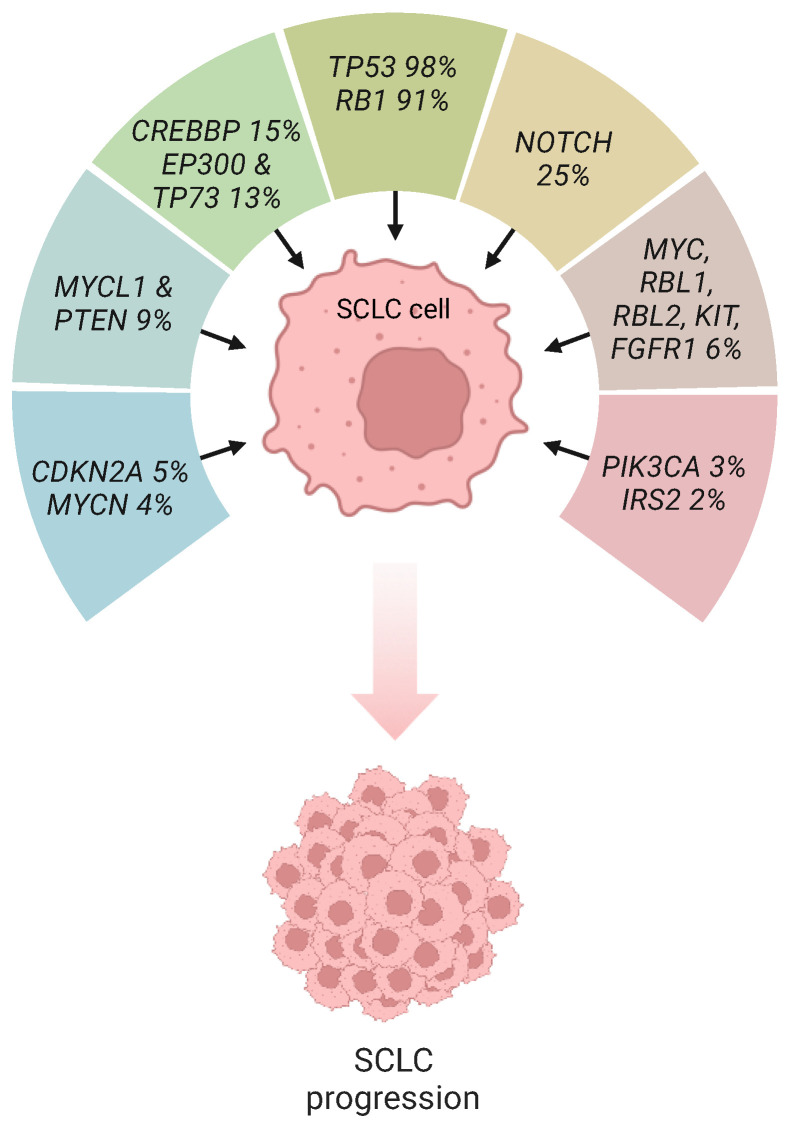
The genomic landscape of SCLC. Percentages refer to the frequency of gene mutations. CDKN2A, cyclin-dependent kinase 2A; CREBBP, CREB binding protein; EP300, E1A binding protein p300; FGFR1, fibroblast growth factor receptor 2; IRS2, insulin receptor substrate 2; MYC, v-myc avian myelocytomatosis viral oncogene homolog; MYCL1, v-myc avian myelocytomatosis viral oncogene homolog 1, lung carcinoma derived; MYCN, v-myc avian myelocytomatosis viral oncogene neuroblastoma derived homolog; PIK3CA, phosphatidylinositol-4,5-bisphosphate 3-kinase catalytic subunit alpha; PTEN, phosphatase and tensin homolog; RB1, retinoblastoma; RBL1, Rb transcriptional corepressor like 1; RBL2, Rb transcriptional corepressor like 2; TP73, tumor protein 73. This figure was created using tools provided by BioRender.com (accessed on 12 February 2024).

Regarding SCLC biology, comprehensive analysis of the genomes of SCLC tumors derived from patients has revealed that most recurrent genetic alterations occur in components of the transcriptional machinery, such as transcription factors and chromatin remodeling complexes. Besides p53 and Rb aberrations, studies have identified recurrent mutations in the Rb-related tumor suppressor genes *Rb transcriptional corepressor like 1* (*RBL1*) and *Rb transcriptional corepressor like 2* (*RBL2*); *cyclin-dependent kinase inhibitor 2A* (*CDKN2A*), the histone-modifying enzymes *E1A binding protein p300* (*EP300*), *CREB binding protein* (*CREBBP*), and *mixed-lineage leukemia* (*MLL*); amplification of *B-cell lymphoma 2* (*BCL2*); members of the Myc family of proto-oncogenes (*MYC*, *MYCN*, *MYCL1*); *SRY-box transcription factor 2* (*SOX2*); *fibroblast growth factor receptor 1* (*FGFR1*); *KIT*; *insulin receptor substrate 2* (*IRS2*); *nuclear factor I B* (*NFIB*); components of the mTOR signaling pathway, including *phosphatidylinositol-4,5-bisphosphate 3-kinase catalytic subunit alpha* (*PIK3CA*), *regulatory associated protein of mTOR complex 1* (*RPTOR*), *RPTOR independent companion of mTOR complex 2* (*RICTOR*), *TSC complex subunit 2* (*TSC2*), and *phosphatase and tensin homolog* (*PTEN*); mutations leading to N-terminal dominant-negative truncations in the *tumor protein 73* (*TP73*); and inactivating mutations in the members of the Notch family of receptors; as well as in Myc-regulatory factors, such as *Myc-associated factor X* (*MAX*), *Max gene-associated protein* (*MGA*), and *Brahma-related gene-1* (*BRG1*) (Figure 1) [3,19]. Lesions in the genes encoding the transcriptional regulators p53 and Rb are present in the majority of SCLC tumors, reaching up to 100% and 93%, respectively [19]. Although other SCLC genomic analyses provide somewhat different mutational frequencies, probably as a result of a different sample size and sequencing technique, all studies have suggested that the loss of both tumor suppressors p53 and Rb is catalytic for the development of SCLC. Missense mutations, chromosomal deletions, and truncating mutations comprise the most frequent genetic aberrations of *TP53* and *RB1* in SCLC. As far as *TP53* is concerned, missense mutations affected the DNA sequence responsible for encoding the DNA-binding protein domain. With respect to *RB1*, several mutations occurred in the DNA sequences located at intron–exon boundaries, thus generating splice-variants that were translated into non-functioning Rb proteins [19].

The fact that both *TP53* and *RB1* genes are ubiquitously inactivated via genetic aberrations in SCLC tumors suggests that elimination of their extensive tumor suppressive functions is an essential step in the development of these aggressive lung cancers. A valuable research tool that has allowed cancer researchers to further understand SCLC biology was the generation and study of a genetically engineered mouse model (GEMM) with somatic inactivation of both p53 and Rb. Meuwissen et al. generated the original SCLC GEMM by lung-specific compound deletion of the *TP53* and *RB1* genes and showed that complete loss of these genes led to the development of SCLC tumors, resembling and recapitulating many characteristics of human SCLC tumors [20]. Interestingly, the authors observed that these *TP53*/*RB1*-mutated mice exhibited a long tumor latent period, lasting approximately 9–12 months after the engineered deletion of *TP53* and *RB1*. This indicates that secondary oncogenic alterations are required for *TP53*/*RB1*-deficient neuroendocrine cells in the lung epithelium to become malignant, an idea which has been validated by the generation of SCLC GEMMs that, in addition to the Cre-mediated deletion of *TP53* and *RB1*, also harbor further genetic aberrations and demonstrate a shorter tumor latent period [21,22,23,24,25,26,27,28]. Furthermore, in corroboration of the above, when *TP53*/*RB1*-null lung epithelial cells were isolated from a SCLC GEMM one month after genetic ablation of both *TP53* and *RB1* genes and cultured in vitro, they only continued to proliferate, without transforming into malignant cells [29]. Based on these findings, we could perhaps consider lung epithelial cells with inactivated p53 and Rb as precancerous SCLC cells that are able to proliferate indefinitely, while simultaneously escaping entrance into cellular senescence and apoptosis. Additionally, loss of Rb seems to confer an additional attribute to these cells, namely the capability of following a neuroendocrine differentiation, since conditional knockout of only the *RB1* gene in lung epithelial cells caused increased proliferation of neuroendocrine cells [30].

In a recent study, Sivakumar et al. performed an integrative genomic analysis of the largest real-world cohort of SCLC tumors evaluated to date, encompassing tumor samples from 3600 SCLC cases [31]. Their data revealed that the most frequent genomic alterations occurred in the *TP53* and *RB1* genes and were present in 91.6% and 73.5% of SCLC cases, respectively. *TP53* inactivation resulted from the generation of non-functioning short variants, due to base substitutions and insertions or deletions (98%), while *RB1* inactivation was brought about mainly via short variants (85%) and to a lesser extent via focal copy-number loss (14%). Gene rearrangements were also found to be responsible for inactivation of both genes, reported in 31 tumor specimens for *RB1* and 8 tumor specimens for *TP53* from a total of 338 tumors with gene rearrangements. An interesting finding of this study, which could potentially be harnessed in the future for the stratification and management of SCLC patients, was the identification of SCLC tumors without genomic inactivation of *TP53* and/or *RB1*. Specifically, the authors observed that in a cohort of 3590 SCLC tumors, 96 were wild-type *TP53* (2.7%), 747 were wild-type *RB1* (20.8%), and 197 were wild-type for both *TP53* and *RB1* (5.5%). In addition, SCLC tumors with *TP53* wild-type or *RB1* wild-type presented a statistically significant correlation with a low tumor mutational burden status, whereas SCLC tumors with both wild-type *TP53* and *RB1* displayed the lowest tumor mutational burden status. The authors also evaluated the co-occurrence and mutual exclusivity of other genetic alterations in SCLC tumors with wild-type *TP53* and/or *RB1* in comparison with *TP53*- and *RB1*-mutant SCLC tumors, and found that loss-of-function mutations in *CDKN2A*, encoding the positive modulator of Rb, namely p16, as well as gain-of-function mutations *CCND1*, encoding the negative modulator of Rb, namely cyclin D1, showed mutual exclusivity with genomic aberrations in *RB1*. In a similar fashion, gaining multiple copies of *MDM2*, whose protein product functions as a negative p53 regulator, was mutually exclusive with *TP53* inactivation. Another observation of this study was the finding that wild-type *TP53* and *RB1* SCLC tumors were enriched for mutations in genes frequently associated with non-small cell lung cancer (NSCLC), including *Kirsten rat sarcoma virus* (*KRAS*), *BRAF*, *Kelch-like ECH-associated protein 1* (*KEAP1*), and *fibroblast growth factor receptor 1* (*FGFR1*). This implies that such SCLC tumors may represent NSCLC that has been transformed into SCLC. From a clinical perspective, in terms of median overall survival, the authors did not find any statistically significant difference between wild-type *TP53*/*RB1* SCLC tumors and *TP53*/*RB1*-mutant SCLC tumors. Similar data were reported for wild-type *RB1* SCLC tumors and *RB1*-mutated SCLC tumors. Nevertheless, SCLC tumors harboring loss-of-function *TP53* mutations exhibited a somewhat lower median overall survival in comparison with SCLC tumors expressing a functioning p53 protein (8.0 vs. 8.8 months, hazard ratio (HR) = 1.6 (1.1–2.5), *p* = 0.03). Finally, a low frequency of genomic alterations in *TP53* or *RB1* was correlated with African genetic ancestry, and *TP53* and *RB1* aberrations were more frequent in older patients compared with younger patients. This study provided valuable data that will trigger the generation of further preclinical and clinical studies, in order to expand our understanding of the molecular underpinnings of SCLC pathobiology and translate this knowledge into better clinical outcomes in patients with SCLC. For example, given that we are now aware of the existence of a small percentage of SCLC patients that carry wild-type copies of *RB1*, together with the fact that SCLC cells with a functional Rb protein may be amenable to CDK4/6 inhibition and more responsive to immune checkpoint inhibitors compared with SCLC tumors with mutated *RB1*, we can design clinical trials with SCLC patients stratified for wild-type *RB1* and evaluate the administration of CDK4/6 inhibitors and immune checkpoint inhibitors individually or in combination with currently available approved drugs for SCLC [32,33].

## 3. Aberrant p53 and Rb Signaling in SCLC

**Figure 2:**
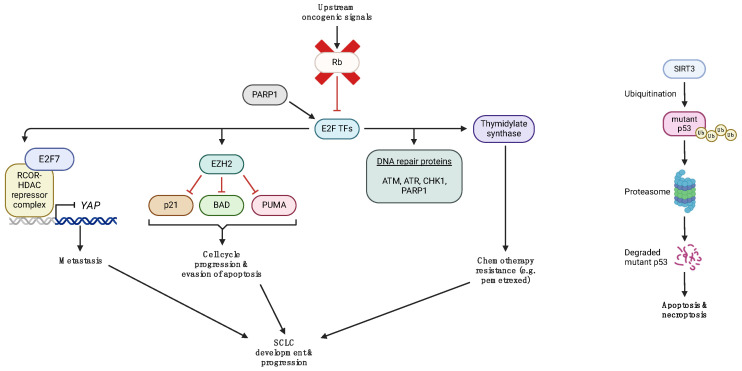
Molecular mechanisms of aberrant p53 and Rb signaling in SCLC. Details are found within the text. The red X sign behind the Rb protein represents Rb inactivation. ATM, ataxia-telangiectasia mutated; ATR, ataxia telangiectasia and Rad3-related protein; BAD, BCL2-associated agonist of cell death; CHK1, checkpoint kinase 1; EZH2, enhancer of zeste homolog 2; HDAC, histone deacetylase; PARP1, poly [ADP-ribose] polymerase 1; PUMA, p53 upregulated modulator of apoptosis; Rb, retinoblastoma; RCOR, REST Corepressor; SIRT3, sirtuin-3; SCLC, small cell lung cancer; Ub, ubiquitin; YAP, yes-associated protein. This figure was created using tools provided by BioRender.com (accessed on 12 February 2024).

As a result of *TP53* and *RB1* inactivation in the vast majority of SCLCs, the upstream and downstream pathways associated with p53 and Rb are disrupted, thereby eliminating their tumor suppressive effects and promoting SCLC development and progression instead (Figure 2). Several preclinical studies have emerged that uncovered the molecular mechanisms of these oncogenic processes and, importantly, suggested novel potential targets that could be utilized for the development of drugs to tackle SCLC via different strategies.

A recent study by Hubaux et al. investigated the functional role of enhancer of zeste homolog 2 (EZH2), a histone-lysine N-methyltransferase that methylates histone proteins and induces transcriptional repression, in the biology of SCLC [34]. By targeting EZH2 with shRNA, the authors probed the effects of EZH2 silencing on the cell cycle and cell death. Their data show that EZH2 blocks apoptosis via downregulating the protein expression of the pro-apoptotic factors p53 upregulated modulator of apoptosis (PUMA) and BCL2 associated agonist of cell death (BAD), as well as promoting cell cycle progression through downregulating the expression of the cell cycle inhibitor p21 [34]. Byers et al. demonstrated that SCLC cells have an increased expression of the transcription factor E2F1 and its target genes, including EZH2, thymidylate synthase, mediators of apoptosis, and DNA repair proteins, such as poly [ADP-ribose] polymerase 1 (PARP1), checkpoint kinase 1 (CHK1), ataxia-telangiectasia mutated (ATM), and ataxia telangiectasia and Rad3-related protein (ATR) [35]. In the setting of NSCLC, thymidylate synthase renders cancer cells resistant to the chemotherapeutic drug pemetrexed, while in patients with SCLC, it may, in part, be responsible for the lower efficacy of the drug combination pemetrexed and carboplatin in comparison with the chemotherapeutic regimen of etoposide plus carboplatin [36,37,38]. In another study, Tang et al. explored the functional relationship between mutant p53 and the NAD-dependent deacetylase sirtuin-3 (SIRT3) in human SCLC cells. They found that SIRT3 triggers the ubiquitination and proteasomal degradation of mutant p53, resulting in the promotion of apoptosis and necroptosis of SCLC cells [39]. Recently, Wu et al. discovered that inactivation of *RB1* in SCLC leads to the repression of *yes-associated protein* (*YAP*) transcription, which, in turn, enhances the metastatic potential of SCLC cells [40]. Mechanistically, loss of *RB1* allows the transcription factor E2F7 to recruit the repressive REST Corepressor 1 (RCOR1/CoREST1)-lysine-specific demethylase 1A (LSD1, KDM1A)-histone deacetylases 1 and 2 (HDAC1/2) complex to the gene promoter of *YAP* and to downregulate its expression. They also found that entinostat, a benzamide histone deacetylase inhibitor, prolonged the survival of a SCLC xenograft model.

## 4. Targeted Therapies in p53 and RB Deficient SCLC Cells

The purpose of unraveling the molecular pathways involved in p53 and RB aberrations in SCLC is to establish effective targeted therapies that will prolong the progression-free survival (PFS) and overall survival (OS) of these patients. Neither TP53 nor RB1 have been therapeutically targetable to date. Thus, efforts are preclinically and clinically oriented towards finding therapies targeting the molecular pathways dysregulated in p53- and RB-deficient SCLC cells (Table 1 and Table 2).

### 4.1. AURK A/B Inhibitors

Overexpression of Aurora kinases (AURKs) is a prevalent protumorigenic pathway in numerous cancer types, including SCLC [41]. In particular, in p53-deficient cells, the protumorigenic effects of the AURKs are even augmented [42]. About 10 years ago, it was found that blocking AURK A or B halts the proliferation and growth of both in vitro and in vivo SCLC models [43]. Alisertib is a selective, small molecule, and orally administered AURK A inhibitor [44]. Its safety and antitumor activity were explored in a prior phase II clinical trial (NCT01045421) of patients with SCLC, breast cancer, NSCLC, head and neck squamous cell carcinoma, and gastroesophageal adenocarcinoma. The encouraging findings provided a rationale for the development of the phase II trial PUMA-ALI-4201 (NCT06095505), which will explore the efficacy of alisertib monotherapy towards extensive stage SCLC patients who have progressed after first-line platinum-based chemotherapy and immunotherapy. Chiauranib is an AURK B inhibitor currently being evaluated in multiple ongoing clinical trials in SCLC patients. A phase III clinical trial (NCT04830813) will explore chiauranib monotherapy in patients who have received at least two different systemic chemotherapy regimens (contained platinum-based regimen) and have progressed or relapsed extensive stage (ES)-SCLC.

Multiple attempts have been made to combine AURK inhibitors with chemotherapy as a treatment option for SCLC. The most recent data are from a randomized phase II trial combining alisertib with paclitaxel as second line therapy in patients with cMYC-positive SCLC. A total of 178 patients with relapsed or refractory SCLC were enrolled, stratified by relapse type (sensitive versus (vs.) refractory/resistant) and the presence of brain metastases. They were randomized 1:1 to alisertib/paclitaxel or placebo plus paclitaxel (89 patients in each group). The promising activity of alisertib/paclitaxel in relapsed or refractory SCLC was confirmed by the 3.2 months median progression-free survival (mPFS) for alisertib/paclitaxel compared to 2.17 months for placebo/paclitaxel (hazard ratio (HR) = 0.77) [45]. Preliminary clinical trials revealed significant indications of AURK inhibitors’ effectiveness, especially when combined with taxanes [41], but these findings require validation through phase III randomized trials.

Recently, there have also been attempts to combine AURK inhibitors with immunotherapy in SCLC. Preclinically, AURK A inhibition blocks SCLC cells in mitosis with restored interferon signaling, promoting T-lymphocyte infiltration [46]. Li et al. demonstrated that AURK A inhibition combined with PD-L1 immunotherapy has long-lasting efficacy in SCLC mouse models [46].

### 4.2. CDK7 Inhibitors

CDK7, a crucial controller of cell-cycle progression, is the best known cell-cycle regulator in SCLC. CDK7 serves as the catalytic core of the CDK-activating kinase (CAK) complex, becoming activated through binding with Cyclin H and Mat1. The trimeric CAK complex activates various core cell-cycle CDKs through phosphorylation [47]. In p53 and RB deficient SCLC murine models, a selective CDK7 inhibitor, YKL-5-12, was tested in association with anti-topoisomerase I, topotecan, and immune checkpoint inhibitors (ICIs). CDK7 inhibition impairs cell cycle and DNA replication and induces genome instability in SCLC cells, also enhancing immune response signaling. In this context, combining YKL-5-124 with anti-PD-1 showed a significant preclinical survival benefit [48]. There is an active phase I study (NCT04247126) on SY 5609, an oral and selective CDK7 inhibitor, in adult patients with advanced solid tumors for which standard curative or palliative measures do not exist or are no longer effective.

### 4.3. DDR Inhibitors

Nearly all cases of SCLC exhibit either homozygous loss or inactivation of RB1, a key regulator of the G1-S cell cycle checkpoint, and TP53, which is essential for multiple DNA Damage Response (DDR) pathways. As a result, SCLC cells exhibit high expression levels of DDR proteins [19,49]. On this basis, numerous inhibitors of DNA repair have recently been created and subjected to assessment in both preclinical models and clinical trials as potential candidates for treating SCLC.

#### 4.3.1. CHK1 Inhibitors

SCLC cell lines possess an elevated level of both CHK1 gene and protein expression [35]. Preclinical efficacy was evoked by targeting the overexpressed cell-cycle checkpoint kinase CHK1 in SCLC cell lines [50]. Prexasertib, a selective CHK1 inhibitor, was explored as a single agent in a phase II trial (NCT02735980) involving patients with previously treated ES-SCLC, but it did not show favorable activity [51]. The inhibitor was given every 14 days to patients who had experienced progression after no more than two prior lines of therapy and with platinum-sensitive (Cohort 1) or platinum-resistant/platinum-refractory (Cohort 2) disease. In Cohort 1 (n = 58), the overall response rate (ORR) was 5.2%, while in Cohort 2 (n = 60), ORR was 0%. Prexasertib did not exhibit sufficient activity to be considered for further development as a monotherapy in ES-SCLC [51].

#### 4.3.2. PARP Inhibitors

PARP1 is highly expressed in SCLC and was initially identified as a potential therapeutic target in SCLC through preclinical models explored by Byers et al. [35]. Several attempts have been made since to exploit the antitumor effects of PARP inhibitors (PARPi) as a monotherapy or in combination with other treatments. Monotherapy with the PARPi olaparib was evaluated as a maintenance treatment for patients with chemoresponsive SCLC in a phase II trial (ISRCTN 73164486, EudraCT 2010-021165-76). However, there was no notable distinction in either PFS or OS between olaparib and placebo [52]. The combination of PARPi with chemotherapy in ES-SCLC was explored by Owonikoko et al. in a phase I/II randomized trial ECOG-ACRIN 2511 (NCT01642251). The first arm received cisplatin-etoposide (CE) along with the PARPi veliparib (CE + V), and the second arm received CE along with placebo (CE + P). The ORR was 71.9% vs. 65.6% for CE + V and CE + P, respectively. The incorporation of veliparib into frontline chemotherapy showed effectiveness in patients with ES-SCLC, and the study achieved its pre-specified endpoint [53]. Another phase II randomized trial (NCT02289690) investigated veliparib plus carboplatin and etoposide in patients with treatment-naïve ES-SCLC. The randomization was 1:1:1: veliparib plus chemotherapy with carboplatin plus etoposide followed by veliparib maintenance, veliparib plus carboplatin plus etoposide followed by placebo, or placebo plus carboplatin plus etoposide followed by placebo (control arm). The combination in the first arm demonstrated an enhancement in PFS compared to the control arm (HR 0.67; 80% confidence interval (CI) 0.50–0.88; *p* = 0.059); however, the difference was not clinically significant (median PFS 5.8 versus 5.6 months), with a trend observed in SLFN11 positive patients (HR 0.6; 80% CI: 0.36–0.97). Additionally, there was no notable advantage of OS [54]. Another PARPi, niraparib, was evaluated as a first-line maintenance therapy in Chinese patients with platinum-responsive ES-SCLC in a phase III randomized trial ZL-2306-005. Although the study did not meet its primary endpoint, niraparib as a maintenance therapy was shown to modestly improve PFS in patients with platinum-responsive ES-SCLC (HR 0.66; 95% CI: 0.46–0.95; *p* = 0.0242), even though not being clinically significant (mPFS 1.54 vs. 1.36 months) [55].

PARP inhibition has also been evaluated in combination with the alkylating agent temozolamide (TMZ). A phase II, randomized, double-blind study (NCT01638546) evaluated whether addition of the PARPi veliparib to TMZ improved 4-month PFS. There was no significant difference in 4-month PFS between TMZ/veliparib (36%) and TMZ/placebo (27%; *p* = 0.19); median OS was also not improved significantly with TMZ/veliparib (8.2 months; 95% CI, 6.4 to 12.2 months; vs. 7.0 months; 95% CI, 5.3 to 9.5 months; *p* = 0.50). Interestingly, ORR was significantly higher in patients receiving TMZ/veliparib compared with TMZ/placebo (39% vs. 14%; *p* = 0.016) [56]. In another phase I/II trial (NCT02446704), TMZ was also combined with olaparib in patients with relapsed SCLC. After a median follow-up of 7.1 months, across all dose levels, the mPFS was 4.2 months (95% CI, 2.8–5.7) and the median OS was 8.5 months (95% CI, 5.1–11.3) [57]. An ongoing phase II study is investigating continuous talazoparib in combination with intermittent low-dose TMZ (NCT03672773) in relapsed/refractory SCLC.

Immunotherapy has also been studied in combination with PARP inhibition. Preclinical findings indicated that combining immune checkpoint blockade with PARP inhibition could be a promising therapeutic approach [58]. PARP inhibition has been shown to enhance the antitumor impact of PD-L1 blockade and increase cytotoxic T-cell infiltration in various immunocompetent SCLC models [59]. Moreover, growing evidence suggests that the induction of PD-L1 expression can occur through mechanisms involving DNA double-strand breaks and cytosolic DNA, including the stimulation of interferon gene-mediated innate immune response [60,61,62]. A single-arm, phase II trial (NCT02484404) evaluated olaparib and durvalumab in patients with relapsed SCLC. Partial or complete responses (ORR 10.5%) were observed in 2 out of 19 evaluable patients, including a patient with EGFR-transformed SCLC [63]. The MEDIOLA phase I/II multicenter open-label and single-arm trial also evaluated olaparib plus durvalumab in relapsed SCLC. Forty patients received olaparib monotherapy (300 mg twice daily) for four weeks, followed by a combination of olaparib (300 mg twice daily) and durvalumab 1500 mg intravenously (IV) administered every four weeks. The observed ORR was 10.5% (95% CI: 2.9–24.8). Despite the study not meeting the primary endpoint of a disease control rate at 12 weeks (28.9%), one patient attained a complete response, and three exhibited a partial response [64]. There are currently several ongoing clinical trials evaluating PARPi along with immunotherapy, as presented in Table 2.

#### 4.3.3. ATM/ATR Inhibitors

ATM and ATR inhibitors have recently been combined with standard chemotherapy regimens for the treatment of SCLC. An interesting approach has been the combination of ATM/ATR inhibitors along with the topoisomerase I inhibitor topotecan, as it exacerbates replication stress in SCLC cells. A phase II trial (NCT02487095) combined M6620, an ATR inhibitor, and topotecan in SCLC patients who had relapsed after at least one prior chemotherapy. Overall, 9 out of 25 patients (36%, 95% CI: 18.0–57.5) achieved a confirmed partial response, meeting the primary endpoint for response. Most patients (17/25 patients; 68%) experienced tumor regressions [65]. Recently, it was preclinically observed that the RNA Pol-II inhibitor lurbinectedin, which induces DNA damage, exhibited strong synergy with the ATR inhibitor berzosertib in SCLC cell lines. Synergy was reduced with high p21 expression, as p21 causes G1 arrest [66]. It could be possible that SCLC cells with p53 aberrations, and thus reduced levels of the protein p21, would respond better to the combination of berzosertib and lurbinectedin. On this basis, an ongoing phase I/II clinical trial (NCT04802174) is examining the combination of lurbinectedin and berzosertib in relapsed SCLC. Several studies are also ongoing regarding SCLC patients with ATM deficiencies (Table 2).

## 5. Conclusions

SCLC cancer is a highly aggressive and lethal lung cancer that is characterized by genetic inactivation of the tumor suppressive genes *TP53* and *RB1*. Preclinical research on SCLC cells and SCLC GEMMs, as well as genomic analyses of SCLC tumors derived from patients, reveal that these two genes are the most frequently mutated and support the notion that the inactivation of p53 and Rb is the catalytic step in the development of SCLC. Aberrations in *TP53* and *RB1* form the setting for further oncogenic events to occur and transform precancerous cells into malignant cells. Although we do not yet possess drugs that are able to revive mutated p53 and Rb, targeting the effects of their loss is a promising therapeutic strategy that is currently being exploited in clinical trials. A major consequence of the loss of *TP53* and *RB1* is the upregulation of the DNA damage repair pathway, and most drug development efforts are now focusing on the inhibition of multiple DNA repair proteins, such as PARP1 and CHK1. PARP inhibitors seem to be a promising therapeutic strategy, as data from preclinical and clinical studies indicate that these agents not only block tumor growth when used as a monotherapy but can also enhance the response to treatment in SCLC by making cancer cells more sensitive to currently available treatment modalities, including chemotherapy, radiotherapy, targeted therapy, and immunotherapy. When combined with other therapies, PARP inhibitors leave very few options, if any at all, for SCLC cells to repair the ongoing molecular damage elicited by other drugs. In order to maximize their activity and realize their full potential in the clinic, future research needs to identify predictive biomarkers for PARP inhibitors and, thus, optimize the selection of patients that will benefit the most from these novel therapies. If we are to fully grasp the functional significance of p53 and Rb aberrations, we need to further elucidate the molecular underpinnings of their intricate pathobiology during SCLC development, which will assist us in developing therapeutic strategies that may halt this deadly malignancy at its initial steps. 

## Figures and Tables

**Table 1:** Completed clinical trials in patients with deficient p53 and RB SCLC.

Agent	NCT ID Number	Phase	Treatment
AURK inhibitors	NCT00858377	I	AMG 900 monotherapy
	n/a	I	Danusertib monotherapy (24 h infusion)
	NCT01045421	I-II	Alisertib monotherapy
	NCT01923337	I	Alisertib + Irinotecan
	NCT01094288	I	Alisertib + Docetaxel
	NCT01677559	I	Alisertib + Nab-paclitaxel
	2006-003772-35	II	Danusertib monotherapy
	NCT02038647	II	Alisertib + Paclitaxel vs. Placebo + Paclitaxel
CHK1 inhibitors	NCT02735980	II	Prexasertib monotherapy
PARP inhibitors	NCT01642251	I/II	cisplatin-etoposide (CE) + Veliparib vs. Placebo + Veliparib
	NCT02446704	I/II	Olaparib + TMZ
	NCT02734004	I/II	Olaparib + Durvalumab
	ISRCTN 73164486, EudraCT 2010-021165-76	II	Olaparib monotherapy
	NCT02289690	II	1:1:1: veliparib + carboplatin + etoposide followed by veliparib maintenance, veliparib + carboplatin + etoposide followed by placebo, placebo+ carboplatin + etoposide followed by placebo
	NCT02484404	II	Olaparib + Durvalumab
	NCT03958045	II	Rucaparib + Nivolumab
	NCT01638546	II	TMZ/veliparib vs. TMZ/placebo
	ZL-2306-005	III	Niraparib monotherapy
ATR inhibitors	NCT02487095	II	Berzosertib + Topotecan
	NCT04768296	II	Berzosertib + Topotecan

ATM, ataxia-telangiectasia mutated; ATR, ataxia telangiectasia and Rad3-related protein; AURK, Aurora kinase; CHK1, checkpoint kinase 1; PARP1, poly [ADP-ribose] polymerase 1; TMZ, temozolomide.

**Table 2:** Ongoing clinical trials in patients with deficient p53 and RB SCLC.

Agent	NCT ID Number	Phase	Treatment Regimen
AURK inhibitors	NCT05271292	Ib/II	Chiauranib
	NCT05505825	Ib/II	AK104 + Chiauranib
	NCT03216343	I	Chiauranib
	NCT04830813	III	Chiauranib Capsule
	NCT06095505	II	Alisertib
CDK7 inhibitors	NCT04247126	I	SY 5609 + gemcitabine
PARP inhibitors	NCT05002868	I	RP12146
	NCT03227016	I	Veliparib + Topotecan
	NCT03532880	I	Olaparib + Low-dose radiotherapy
	NCT03923270	I	Radiotherapy + durvalumab vs. durvalumab combinations (tremelimumab or olaparib)
	ES-SCLC-2nd-IIT-SHR3162-APA	n/a	Camrelizumab + Fluzoparib
	NCT04644068	I/II	AZD5305 monotherapy or in combination with anti-cancer agents
	NCT04826341	I/II	Sacituzumab Govitecan + Berzosertib
	NCT04209595	I/II	PLX038 (PEGylated SN38) + Rucaparib
	NCT04728230	I/II	Carboplatin + etoposide + durvalumab + olaparib and/or radiation therapy
	NCT03830918	I/II	Niraparib + Temozolomide + Atezolizumab
	NCT05975944	I/II	Olaparib + Selinexor
	NCT02769962	I/II	EP0057 + Olaparib
	NCT04434482	I/II	IMP4297 + Temozolomide
	NCT04400188	I/II	Fluzoparib + Temozolomide ± SHR-1316
	NCT04659785	I/II	Fluzoparib + Apatinib
	NCT04538378	II	Olaparib + Durvalumab
	NCT05411679	II	EP0057 + Olaparib
	NCT05718323	II	Niraparib + Anti-PD-L1 therapy
	NCT04334941	II	Talazoparib + Atezolizumab
	NCT05162196	II	Radiotherapy + Niraparib + Toripalimab
	NCT04701307	II	Niraparib + Dostarlimab
	NCT03672773	II	Talazoparib + low-dose Temozolomide
	NCT02498613	II	Cediranib + Olaparib
	NCT05623319	II	Olaparib + Pembrolizumab
	NCT05245994	II	Olaparib + Durvalumab
	NCT04939662	II	Olaparib + Bevacizumab
	NCT04624204	III	Pembrolizumab + Chemoradiation followed by pembrolizumab ± Olaparib vs. Pembrolizumab + Chemoradiation
	NCT04790955	Observational	SBRT and low-dose radiotherapy + PARPi + temozolomide + PD-1/PD-L1 inhibitors
ATR inhibitors	NCT04491942	I	Elimusertib + chemotherapy (cisplatin, or cisplatin and gemcitabine)
	NCT02595931	I	Berzosertib + Irinotecan hydrochloride
	NCT04802174	I/II	Berzosertib + Lurbinectedin
	NCT04826341	I/II	Berzosertib + Sacituzumab govitecan
	NCT02487095	I/II	Berzosertib + Topotecan
	NCT03896503	II	Berzosertib + Topotecan
ATM inhibitors	NCT04939662	II	Olaparib + Bevacizumab

ATM, ataxia-telangiectasia mutated; ATR, ataxia telangiectasia and Rad3-related protein; AURK, Aurora kinase; CDK7, cyclin-dependent kinase 7; ES-SCLC, extensive-stage small cell lung cancer; N/a, not applicable; PARPi, poly [ADP-ribose] polymerase 1 inhibitors; PD1, Programmed cell death protein 1; PDL1, programmed death-ligand 1; SBRT, stereotactic body radiation therapy.

## Data Availability

Data are contained within the article.
